# Management of pacemaker lead thrombus

**DOI:** 10.1016/j.hrcr.2024.08.024

**Published:** 2024-08-30

**Authors:** Michael Jorgensen, Arjun Gajulapalli, David T. Zhang, Roger Fan, Ibrahim O. Almasry, Eric J. Rashba

**Affiliations:** 1 Department of Internal Medicine, Stony Brook Medicine, Stony Brook, NY; 2 Division of Cardiology, Department of Medicine, Stony Brook Medicine, Stony Brook, NY

**Keywords:** Pacemaker lead-associated intracardiac thrombus, Vacuum-assisted thrombectomy

**Key Teaching Points**

• Patients with large or symptomatic thrombi should be initiated on anticoagulation with either a direct oral anticoagulant or warfarin in the absence of contraindication and with infectious etiology ruled out. Bleeding and symptom resolution should be closely monitored, with consideration of lead extraction or alternative therapies should the patient have no response to anticoagulation.
• Transesophageal echocardiogram should be completed prior to planned vacuum-assisted thrombectomy for evaluation of potential risk, including paradoxical embolism, to better characterize clot burden and location.
• Vacuum-assisted thrombectomy offers a potential solution for large intracardiac pacemaker lead thrombi for patients who are poor surgical candidates and are intolerant of systemic anticoagulation. The advent of pulmonary embolism response teams within the past few years further necessitates the importance of having a wide range of available therapies to treat this potentially life-threatening condition.

## Introduction

The prevalence of intracardiac thrombi on transvenous leads from cardiac implantable electronic devices varies widely in the literature from 1.4% to 30%.^1^ Many of these thrombi are found incidentally during lead extraction or on intracardiac echocardiography during ablation procedures.^1^ The following available interventions are described in case reports: surgical embolectomy during cardiopulmonary bypass, mechanical thrombectomy with interventional radiology, pharmacologic thrombolysis, simple lead removal, or systemic anticoagulation.^2^, ^3^, ^4^, ^5^, ^6^ Anticoagulation therapy is the mainstay of treatment for most intracardiac pacemaker lead thromboses.^5^^,^^7^ In this case, we report the successful use of transesophageal echocardiogram (TEE)-guided percutaneous vacuum-assisted thrombectomy (VAT) in a complex patient with extensive comorbidities after failing systemic anticoagulation therapy.

### Case report

A woman aged in her 80s with hypertrophic obstructive cardiomyopathy, status post alcohol septal ablation (2015) complicated by atrioventricular block, status post dual-chamber permanent pacemaker; heart failure with preserved ejection fraction; nonobstructive coronary artery disease; essential thrombocytosis (JAK2^+^); recurrent deep vein thrombosis, status post inferior vena cava filter; and chronic kidney disease stage 3 was admitted with shortness of breath and lower extremity edema. The initial presentation was notable for no hypoxia on room air, but a respiratory rate of 36 breaths/min; all other vital signs were within normal limits.

**Figure 1:**
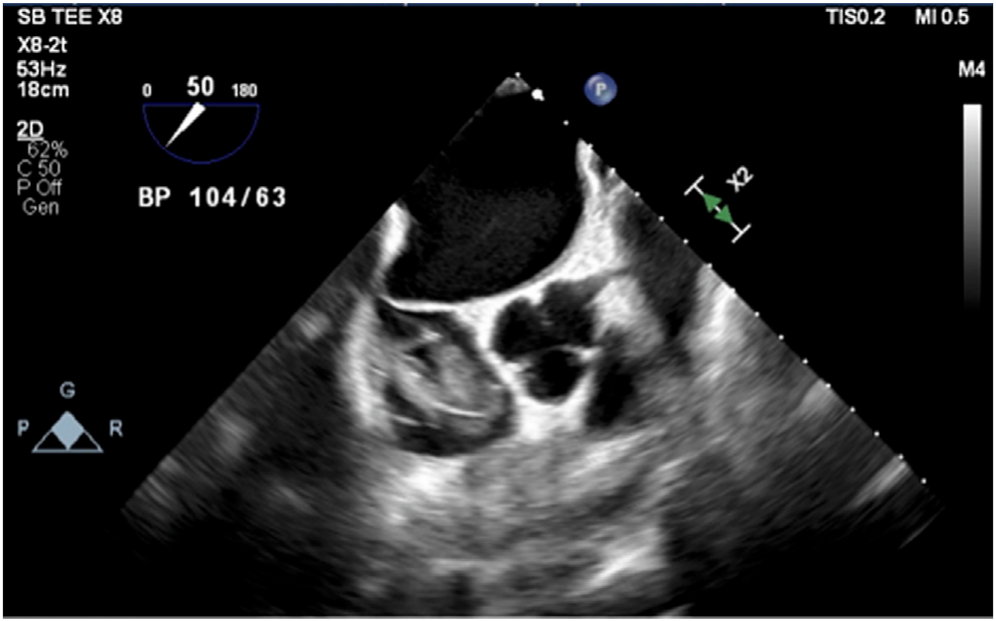
Transesophageal echocardiogram demonstrating a right atrial lead-associated thrombus.

Initial workup was notable for chest radiograph with possible congestion, computed tomography pulmonary angiogram negative for pulmonary embolism, pro–B-type natriuretic peptide 24,689 pg/mL (reference range ≤300 pg/mL), and high-sensitivity troponin peak of 65 ng/L (reference range ≤14 ng/L). Blood cultures throughout her hospitalization were negative for growth, and the patient remained without fever or infectious symptoms. She was given furosemide intravenously for presumed acute decompensated heart failure and admitted to the cardiac telemetry service for further management. Transthoracic echocardiogram (TTE) demonstrated a 5-cm × 2-cm echo density in the right atrium (RA) and prolapsing into the right ventricle, possibly attached to the RA lead. TEE confirmed the thrombus was attached to the RA lead (Figure 1). TEE also demonstrated a patent foramen ovale by 2-dimensional imaging, predominately left to right shunting by color Doppler, with absence of agitated bubbles in the left heart. The permanent pacemaker was interrogated with 23% atrial pacing and no ventricular pacing.

She was started on intravenous heparin without bolus and subsequently developed hypotension. TTE showed a new moderate pericardial effusion and early tamponade physiology. She underwent pericardiocentesis with 400 mL of bloody fluid drained.

**Figure 2:**
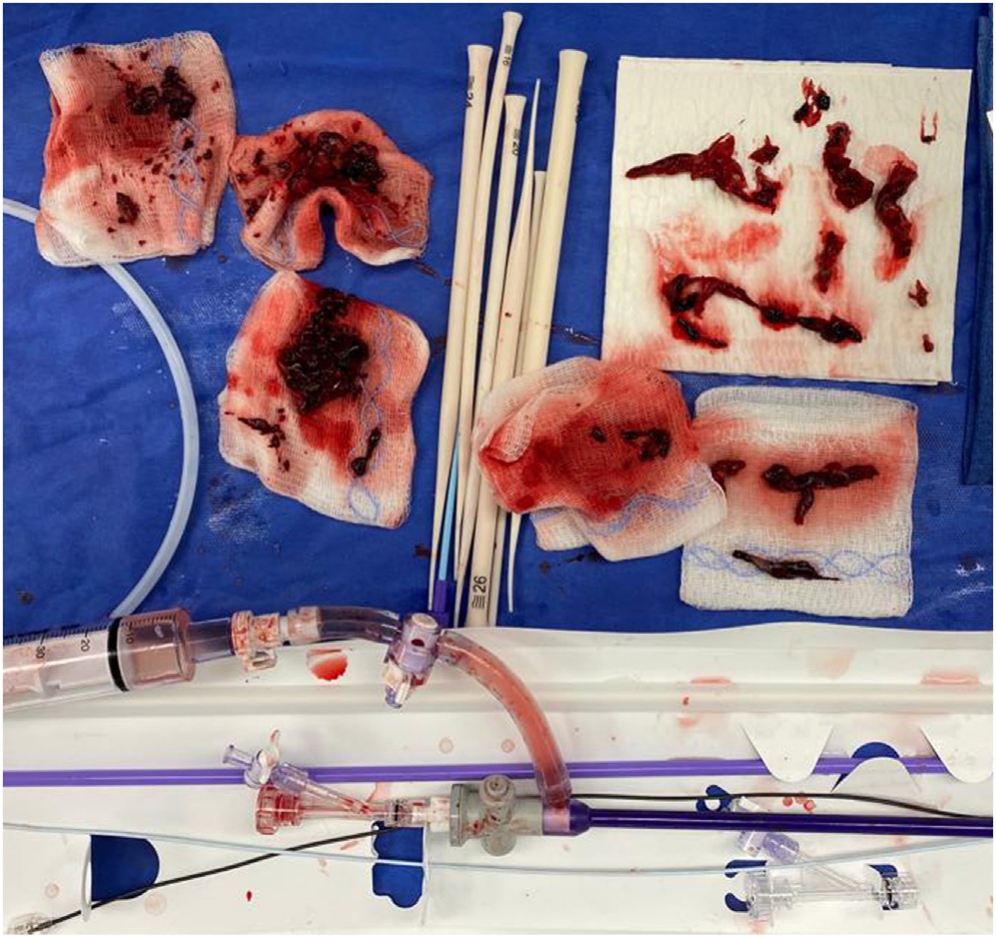
Large clot burden removed using the vacuum-assisted thrombectomy device.

Given the need for anticoagulation resulting in a hemorrhagic effusion, a multidisciplinary discussion was held with electrophysiology, cardiac surgery, and interventional radiology about management options. The patient underwent successful mechanical thrombectomy with interventional radiology. Interprocedurally, a T24 mechanical aspiration device and T20 curve were advanced coaxially into the RA under fluoroscopy. Under TTE guidance, the catheter was positioned immediately adjacent to the hypermobile clot and mechanical thrombectomy performed with a total of 10 aspirations. A large burden of clot within the RA and ventricle was removed with a small burden of residual clot attached to the RA pacemaker lead (Figure 2). Anticoagulation with intravenous heparin drip was resumed after clot aspiration, without hemorrhagic effusion recurrence.

The patient initially improved thereafter, but over the next 2–3 weeks she eventually developed progressive renal failure with anuria. She and her family declined hemodialysis. The patient ultimately expired.

## Discussion

There are no current consensus guidelines for the management of intracardiac pacemaker lead thrombi.^7^ Mobile thrombi on transvenous leads are found commonly in patients referred for lead extraction or ablation procedures.^1^^,^^8^ European Society of Cardiology guidelines for cardiac pacing^9^ state that patients with large or symptomatic thrombi should be initiated on anticoagulation with either a direct oral anticoagulant or warfarin. Bleeding and symptom resolution should be closely monitored, with consideration of lead extraction or alternative therapies should the patient have no response to anticoagulation. TEE is preferred for initial thrombus assessment, with follow-up TEE or TTE completed in 3 months to evaluation for thrombus persistence. Unfortunately, the patient developed a hemorrhagic pericardial effusion with clinical tamponade physiology while on intravenous heparin,^9^^,^^10^ Extraction of leads with incidental <2-cm mobile thrombi has been shown to be safe without associated events.^11^ European Heart Rhythm Association 2020^12^ consensus guidelines suggested thrombi >2 cm be considered for open lead extraction. In rare cases, large lead-associated thrombi have been found to impede pacemaker function, as described by Xia and colleagues^13^ in a patient with appropriate pacing signal but absent QRS complex. The large size of this patient’s pacemaker lead thrombus, extending from the RA into the right ventricle, precluded pacemaker lead removal due to concern for catastrophic pulmonary embolism. Of note, the patient’s pacemaker leads were more than a decade old, increasing the likelihood of subsequent ossification and difficulty with lead removal.^14^ Considering the patient’s limited functional ability, age, and comorbidities, she was deemed high risk for surgical management.

VAT offers a solution to these difficult-to-manage pacemaker lead–associated intracardiac thrombi. In a case report, Mahajan and colleagues^6^ detailed a woman aged in her 50s who underwent successful AngioVac (Angiodynamics, Latham, NY)-guided removal of lead vegetations and ultimately device removal. Further literature has described the role for VAT with infectious lead vegetations, potentially avoiding lead extraction.^3^^,^^15^, ^16^, ^17^, ^18^ Data involving VAT for noninfectious pacemaker lead thrombi are sparse. Yu and colleagues^19^ described a case of a 79-year-old man who initially presented with a deep vein thrombus and pulmonary embolism that eventually evolved into clot in transit with attachment to the right atrial lead; ultimately, after a multidisciplinary effort, interventional radiology was able to aspirate the right atrial thrombus with AngioVac and successfully place an inferior vena cava filter. VAT is likely of greatest benefit for newly formed clots, with less likelihood of calcification.^20^ The advent of pulmonary embolism response teams within the last few years further necessitates having a wide range of available therapies to treat this potentially life-threatening condition.^19^^,^^21^

Fortunately, our patient had successful evacuation of a majority of her clot burden using this technique, with subsequent symptom improvement. Further data are needed to explore the long-term outcomes in patients who undergo VAT, especially with varying degrees of clot chronicity. The evolving role of pulmonary embolism response teams and advancement of noninvasive therapies will continue to expose a wider patient population to VAT. Overall, this case highlights the importance of tailoring therapeutic intervention to patient-specific factors to mitigate the greatest chance of patient harm.

## Disclosures

The authors have no conflicts of interest to disclose.
